# Automated drug dispensing systems in the intensive care unit: a financial analysis

**DOI:** 10.1186/s13054-015-1041-3

**Published:** 2015-09-09

**Authors:** Claire Chapuis, Pierrick Bedouch, Maxime Detavernier, Michel Durand, Gilles Francony, Pierre Lavagne, Luc Foroni, Pierre Albaladejo, Benoit Allenet, Jean-Francois Payen

**Affiliations:** Pôle Pharmacie, CHU Grenoble, Hôpital Michallon, CS 10217, F-38043 Grenoble, France; Université Grenoble Alpes/CNRS, ThEMAS TIMC UMR 5525, Grenoble, F-38041 France; Pole Anesthésie-Réanimation, Hôpital Michallon, Grenoble, F-38043 France

## Abstract

**Introduction:**

To evaluate the economic impact of automated-drug dispensing systems (ADS) in surgical intensive care units (ICUs). A financial analysis was conducted in three adult ICUs of one university hospital, where ADS were implemented, one in each unit, to replace the traditional floor stock system.

**Method:**

Costs were estimated before and after implementation of the ADS on the basis of floor stock inventories, expired drugs, and time spent by nurses and pharmacy technicians on medication-related work activities. A financial analysis was conducted that included operating cash flows, investment cash flows, global cash flow and net present value.

**Results:**

After ADS implementation, nurses spent less time on medication-related activities with an average of 14.7 hours saved per day/33 beds. Pharmacy technicians spent more time on floor-stock activities with an average of 3.5 additional hours per day across the three ICUs. The cost of drug storage was reduced by €44,298 and the cost of expired drugs was reduced by €14,772 per year across the three ICUs. Five years after the initial investment, the global cash flow was €148,229 and the net present value of the project was positive by €510,404.

**Conclusion:**

The financial modeling of the ADS implementation in three ICUs showed a high return on investment for the hospital. Medication-related costs and nursing time dedicated to medications are reduced with ADS.

## Introduction

Reducing health care costs has become a critical concern for hospitals. Pharmacists are implementing methods to provide safety and efficiency in the medication process. Optimization of drug delivery through automated drug dispensing systems (ADS) may be valuable in hospital departments with an uncontrolled floor stock [1]. Automation has also the potential to free pharmaceutical staff and nurses from the time-consuming process of distributing and preparing medications. Patients admitted to the intensive care unit (ICU) are particularly susceptible to medication errors and adverse events due to alterations in the pharmacokinetics of drugs, the intravenous administration of drugs with a narrow therapeutic index, and the high turnover in medications [2, 3]. The estimated annual costs attributable to preventable adverse drug events for a 700-bed teaching hospital were about $2.8 million [4]. Implementing ADS in the ICU could optimize floor stock management and also reduce costs related to medication errors. Studies focusing on the economic impact of ADS are few and most were published in the 1990s in the United States [1, 5–7]. One Canadian report has already shown a reduction of $152,000 in the cost of inventory stocking and allocated nurses’ time along with more than 25 % decrease in error rates [8]. The same report [8] and a recent Canadian review [9] recommended that informed decision-making about the use of ADS required an assessment of the clinical and economic consequences of adoption. While a recent survey reported an implementation rate for ADS of 89 % in the United States [10], there is a lack of European data. In France, only a few hospitals have implemented them. In our hospital, an ADS was implemented in one pilot surgical ICU in 2005, and then in one medical ICU. We found that ADS markedly decreased the rates of administration of medication errors in the medical ICU [11]. Thereafter we completed the implementation of ADS across three adult surgical ICUs in 2011, namely neurosurgical, cardiac and trauma ICUs. The aim of the present study was to evaluate the economic impact of ADS in three surgical ICUs using a cash flow analysis.

## Methods

### Population and setting

This study was conducted at the University Hospital of Grenoble (France) in three surgical ICUs each with 12, 12 and 9 beds, respectively, and a total of 2,082 admissions and 11,327 patient-days in 2011. Before the implementation of ADS, each ICU had an open floor stock drug-distribution system. Medications were delivered through boxes to the ICUs every weekday by pharmacy technicians and by pharmacists at nights and weekends in the case of emergency. Before ADS implementation, nurses received boxes of medications on the ward and refilled the medicine cabinet daily. Nurses were responsible for inventory management, restocking, checking for expired drugs and acquiring missing medications. If a medication was unavailable, nurses were expected to retrieve it from another location. An unsuccessful search meant that a caregiver needed to go to the central pharmacy.

### Intervention

An OmniRx ADS (Omnicell, Mountain View, CA, USA) was implemented in each ICU. Computer-controlled ADS meant that most of the medications were stored directly in the care unit and each drug delivery was recorded. The cabinets were locked, and their access was gained through passwords or fingerprint identification. These cabinets used a non-profiled mode, in which medication orders were not entered either manually or electronically into the pharmacy information system. Nurses accessed all medications stored in the cabinet. After they had selected the patient name, nurses selected prescribed medications from the global list of medications for this patient. The ADS computer recorded the time, the nurse identity, the patient name and the medications removed. For narcotic medications, they also had to enter the physician name. After implementation, the ADS compartments were reloaded daily on the wards by a pharmacy technician. Pharmacy technicians had access to the interfaced pharmacy information system to determine which drugs needed reloading. Pharmacy technicians could use the inventory report to check expiration dates. With the ADS up to 250 usable medications were stored in the unit, 50 % less than the number stored in the medicine cabinet before the intervention. The storage aims were to maintain at least 90 % of each unit’s formulary. Some medications were ordered only for a single patient (i.e., specific antiepileptic treatment). Once the patient had been discharged, these medications were returned by the pharmacy technician on notification by the nurse, instead of being stored in the cabinet. The implementation of the ADS in each ICU was associated with a training program for nurses, including basics for use, and prevention and correction of medication errors. The opportunities for errors that were discussed with nurses in these interviews were selecting the wrong patient, wrong choice of medication, and wrong dose and narcotics tracking. The ADS implementation was accompanied daily by a pharmacist and a pharmacy technician.

### Cash flow analysis

The cost of implementing the ADS in the three ICUs was calculated as follows: 1) the cost of ADS including the development of the software interface between the pharmacy department and the ADS computer system; 2) the cost of drug storage estimated on the basis of floor stock inventories and outdated drugs; and 3) the time spent by nurses and pharmacy technicians on medication-related work activities before and after implementation of ADS, as assessed with a work-sampling method by an independent evaluator during 40 day shifts (20 nurses and 20 pharmacy technicians). Direct observations were performed by a pharmacy student from 8:00 a.m. to 5:00 p.m. over a period of 10 days, one month prior and one month after the ADS implementation. The observer had been introduced previously to the nursing staff, in order to minimize the “Hawthorne effect”. Observed nurses and pharmacy technicians implicitly agreed to participate to this monitoring by an external observer*.* The observer used a predefined classification of activities. Medication-related work activities were related to collection and preparation of drugs, inventorying and stocking of medications, removing expired drugs, and filling and checking drug boxes or exchange carts and travel time between the pharmacy and the units. Time assessment was performed based on 7 working days for nurses and 5 working days for pharmacy technicians (who did not work on the weekends). The average hourly labor cost for nurses and technicians was €25 (in French public hospitals, nurses and pharmacy technicians have the same salary according to the French hospital salary scale).

Cash flow (CF) analysis is a commonly used method to assess a financial investment and determine the robustness of a project. For this analysis, we considered the global cash flow to represent the value of generated cash to pay back the invested capital and the net present value (NPV) as the present value of the cash inflows minus that of the cash outflows, at a specified average annual rate of return, according to the formula [12]:

$$ \mathrm{N}\mathrm{P}\mathrm{V}\ \left(\mathrm{t},\ \mathrm{C}{\mathrm{F}}_{\mathrm{t}}\right) = \Sigma\ \left(\mathrm{n},\ \mathrm{t}=0\right)\ \mathrm{C}{\mathrm{F}}_{\mathrm{t}}/\ {\left(1+\mathrm{i}\right)}^{\mathrm{t}} $$

where t is the period, CF_t_ is the cash flow during that period, n is the total number of periods and i is the rate of return. The NPV represents the project value, when the capital cost to the decision maker is given. If the NPV value is positive, the project is financially profitable.

We conducted a cash flow analysis considering the implementation of the ADS as a financial investment. The hourly indexed labor cost was 2 % according to the French inflation rate in 2011. Operating cash flows (time gain, eviction of expired drugs), investment cash flows (ADS, software and storage reduction), global cash flows resulting from operating and investment cash flows over a 5-year period, and NPV at a 4 % annual rate of return were evaluated. The annual rate of return of 4 % was equal to the long-term loan rate of hospitals in 2011. The residual cost of the ADS at year 5 was estimated to be 10 % of the initial price.

In addition, we measured the incidence of missing medications prior to and after implementation of ADS in the three ICUs over a period of one month each. This study did not concern patients, therefore was not subject to the need for medical ethical approval, and no consent was needed.

## Results

The equipment cost included the ADS (€40,500 each) and the development of interface software between the computer systems in pharmacy and the ADS (€4,500) (Table 1). The cost of drug storage was €93,832 before implementation of the ADS and €49,525 after. In addition, the cost of expired drugs was lowered by €14,772 each year. We found that expired drugs were completely eliminated with the ADS due to a rotation of drug stocks and the regular monitoring of expiration dates.

**Table 1:** Cash flow analysis of the implementation of the automated dispensing system (ADS) in three ICUs

	Year 0	Year 1	Year 2	Year 3	Year 4	Year 5
	(2011)	(2012)	(2013)	(2014)	(2015)	(2016)
*Operations*						
Nurse time gained (€)	/	133,833	136,510	139,240	142,025	144,865
Pharmacy technician additional cost (€)	/	−22,750	−23,205	−23,669	−24,142	−24,625
Saving from reduced outdated drugs (€)	/	14,772	15,067	15,369	15,676	15,990
Maintenance costs (€)	0	333	0	4832	2743	/
Total (operating cash flow) (€)	/	126,188	128,372	135,772	136,302	136,229
*Investment*						
ADS (€) in three ICUs	−121,500	/	/	/	/	12,000
Software (€)	−4,500	/	/	/	/	/
Saving from reduced drug storage (€)	44,298	/	/	/	/	/
Total (investment cash flow) *(€)*	−81,702					12,000
*Global cash flow (€)*	−81,702	126,188	128,372	135,772	136,302	148,229

After implementation of the ADS, nurses spent less time doing pharmacy-related activities, with a mean time gain of 14.7 hours per day for the three ICUs (4 hours/9 beds). With 33 beds and a nurse-to-bed ratio of 2, this time gain was identified as −2.9 hours/day to collect the medications, −9.8 hours/day to prepare individual doses, −1.8 hours/day to order and stock medications and – 0.2 hours/day to remove expired drugs.

Pharmacy technicians spent more time doing floor-stock activities, with a mean additional 3.5 hours per day for the three ICUs: −0.74 hours/day to prepare boxes, +1.72 hours/day to prepare medications in exchange carts for restocking the ADS, + 2.52 hours/day to travel from the pharmacy to the unit and to restock on the ward.

The NPV at 4 % was positive by +€510,404, and global cash flow at 5 years was estimated at €148,229. We found that implementation of ADS was associated with a reduction in the number of missing medications from 84 to 37 (56 %). Missing medications prior to use of the ADS were primarily related to antibiotics and neuromuscular relaxant drugs, while they were related to occasional treatments, e.g., alfuzosine or ropinirole, after implementation of the ADS.

## Discussion

The implementation of ADS in three ICUs was a financially profitable project. Using reasonably estimated hourly indexed labor costs and annual rate of returns, the NPV was markedly positive. An estimated €148,229 global cash flow at 5 years after the initial investment indicated that the implementation would be paid back at this stage.

As we previously said, there are few data on the economic impact of ADS, especially in European countries. This study contributes to a poorly researched aspect of pharmacy practice in a European hospital setting. According to an annualized analysis on non-profiled devices conducted by the Canadian Agency for Drugs and Technologies in Health, savings of approximately $34,000 per patient care unit can be made. After discounting and adjusting for inflation, they estimated net savings of $152,000 per patient care unit over a 5-year period. Overall, a 400-bed hospital would be expected to achieve 5-year savings of $2.7 million with the use of such equipment [8]. That report emphasized that most studies did not report nurse and pharmacy technician time, and their costs, to make appropriate comparisons [8]. Using a work-sampling method, we found that nurses spent significantly less time in ordering, restocking, searching for and picking up medications, with a mean spared time of 14.7 hours per day for the three ICUs. Meanwhile, pharmacy technicians spent an additional 3.5 hours per day managing the ADS stocks. It should be noted, however, that the work-sampling method is an indirect measure of time and provides only an estimate of the time spent performing different activities.

A previous financial analysis on a 36-bed cardiovascular surgery unit and an 8-bed cardiovascular ICU showed that ADS could save the institution about $1 million over 5 years if the personnel time savings could be translated into full-time equivalent reductions [6]. In our institution, the time spent by pharmacy technicians in floor-stocking activities, i.e., 3.5 hours per day, is a cost to the three ICUs. Although no full-time equivalent reduction in nurses was undertaken simultaneously, the spared time for nurses was believed to perform more patient-care-related activities and to manage more appropriately sustained drug storage. According to this analysis, the cost savings associated with ADS were driven largely by reductions in nurses’ time. This is a limitation to our work, in which the potential positive financial effect may be overestimated. We hypothesized that benefits in reducing nursing time spent on logistical activities would be turned into benefits for the organization, including care quality and safety. As it has been previously discussed, conventional economic or financial approaches such as ours do not evaluate quality of care, potential increased healthcare provider productivity or satisfaction.

The number of missing medications was markedly reduced after ADS implementation. The time spent out of care is reduced for nurses and timely treatments are available for the patients. In addition, any situation in which a nurse could not access critical medication from the ADS during an emergency has not yet been reported. User satisfaction has been assessed previously [11, 13]. Overall satisfaction was found to improve after a few months to overcome the “resistance to change” phenomenon [11]. With such a system, pharmacy technicians can become more involved on the ward. The ADS facilitates medication distribution allowing pharmaceutical staff and nurses to focus on tasks defining their specific duties and distinguishable roles. Such efforts should be promoted to improve nurses’ working conditions, considering that fatigue, stress, workload and untrained staff have be shown to be risk factors for medication errors [14]. We could further reduce both time and medication errors if the ADS was interfaced with our computerized order physician entry (CPOE) [15]. Another advantage offered by ADS is improved communication between nurses and pharmaceutical staff [1, 5, 7, 16]. ADS can be considered as a “boundary object” between nursing and pharmaceutical staff. Yet in two satisfaction surveys, the ADS was given high marks by the nurses, and 80 % and 96.7 % of nurses, respectively, wanted to keep the system on their unit [5, 11]. ADS implementation and formulary adjustments may also increase the collaboration between ICU physicians and pharmacists. On-ward participation of a pharmacist was associated with improved quality and efficiency of care [17, 18].

The implementation of new technologies such as ADS can be viewed as part of a global re-organization process, involving a multidisciplinary team including physicians, nurses, pharmacists, unit directors and managers. This approach to the management of medications in ICU may improve efficiency, outcome and the cost of care for critically ill patients [19].

## Conclusion

ADS investment is financially profitable and improves the efficiency of drug distribution. Our results might help managers to consider implementing ADS in their ICUs in hospitals with similar operating structures.

## Key messages

- In this single center before-after study, ward-based ADS reduced costs in the adult surgical ICU
- Economic evaluations of ADS, including nurse and pharmacy technician work-time, are incomplete
- Our study shows that ADS investment is a financially profitable project in the ICU
- ADS can reduce medication-related costs and nursing time dedicated to medications

## Abbreviations

ADS: Automated dispensing system
ICU: Intensive care unit
CF: Cash flow
NPV: Net present value
CPOE: Computerized order physician entry
